# COVID-19 Pandemic Influence on Diabetes Management in Croatia

**DOI:** 10.3389/fcdhc.2021.704807

**Published:** 2021-03-21

**Authors:** Ivan Cerovečki, Marija Švajda

**Affiliations:** ^1^ Division for Public Health, Croatian Institute of Public Health, Zagreb, Croatia; ^2^ Division for Health Informatics and Biostatistics, Croatian Institute of Public Health, Zagreb, Croatia

**Keywords:** COVID-19, CroDiab, diabetes regulation, hospitalizations, primary healthcare

## Abstract

**Aim:**

The study aims to investigate the effects of the COVID-19 pandemic on diabetes management and diabetes patients’ healthcare utilization patterns in Croatia.

**Methods:**

Using data contained in the Croatian diabetes registry (CroDiab), Central Health Information System of the Republic of Croatia (CEZIH), and the Croatian hospitalization database (BSO), indicators including the total number of registered diabetes patients, number of newly diagnosed diabetes cases, number of diabetes-related primary care visits and hospitalizations, and key diabetes control indicators were analyzed. Yearly values from 2017 until 2020 were compared.

**Results:**

The age-adjusted prevalence rate increased significantly from 2017 until 2019 (2017: 6,858/100,000; 2018: 7,053/100,000; 2019: 7,160/100,000). In 2020 the age-adjusted prevalence rate was 7,088/100,000, but the decrease was insignificant compared to 2019. The age-adjusted rate of new cases decreased from 2017 until 2019 (2017: 910/100,000; 2018: 876/100,000; 2019: 845/100,000), with a significant decrease in 2020 (692/100,000) compared to 2019. The number of diabetes panels increased from 2017 (117,676) to 2018 (131,815), with a slight decrease in 2019 (127,742) and a sharp decrease in 2020 (104,159). A similar trend was observed regarding the numbers of diabetes patients with panels, visits to primary healthcare providers for diabetes-related problems and diabetes patients who visited their primary healthcare provider. A slightly different trend was observed regarding diabetes-related hospitalizations. In 2017 there were 91,192 diabetes-related hospitalizations; the number decreased to 83,219 in 2018, increased again to 102,087 in 2019 and decreased to 85,006 in 2020. The number of hospitalized diabetes patients displayed a similar tendency.

**Conclusion:**

The COVID-19 pandemic has had a negative effect on the utilisation of healthcare by diabetes patients, which may have long-term consequences for their general health.

## Introduction

Since its beginning in late 2019 in the People’s Republic of China, the COVID-19 pandemic has been causing major disruptions to healthcare systems worldwide, regarding both healthcare providers and healthcare users (1). Significant numbers of patients in need of hospital treatment for intermediate or severe clinical forms of COVID-19 necessitated the diversion of financial, technological and human resources to intensive care and associated stationary care units, whereas patients were advised to avoid seeking medical care unless it was strictly necessary, leading to irregular provision of most routine regular medical services (2). An analysis conducted by the Health Foundation determined a sharp decrease in the number of consultations, referrals, vaccinations and other primary healthcare use indicators during the initial lockdown imposed during the spring 2020 in the United Kingdom (3). Another study of the impact of the COVID-19 epidemic on the follow-up and control of chronic diseases conducted in Spain found a significant reduction in quantitative healthcare quality indicators during the same period in Catalonia (4). Urgent medical care was found to be affected in some countries as well, as studies conducted in the United States, Italy, Spain and Hong Kong reported decreases of the number of hospitalizations related to urgent cardiologic and neurologic conditions with concurrent increases in respective mortality and complication rates (5–9).

The first case of COVID-19 in Croatia, confirmed by real-time polymerase chain reaction testing, was reported on February 25, 2020. By December 31, 2020, 210,837 cases of COVID-19 were recorded in Croatia with 3,920 deaths and a case-fatality ratio of 1.9% (10). Besides posing a burden on the hospital system, the COVID-19 epidemic also compromised the provision of primary healthcare, as indicated by primary healthcare reports published by the Croatian Institute of Public Health. A significant decrease in the number of contacts, examinations and consultations with primary healthcare providers was recorded in the spring months (March-June) of 2020 during the initial lockdown imposed by Croatian health authorities; the number of patients seeking primary healthcare services increased thereafter during the autumn months (September-December) of 2020, coinciding with the peak of second wave of the COVID-19 epidemic (11, 12).

The prevalence of diabetes mellitus (DM) in Croatia had been estimated by previous research at 8.9% of the adult population, although recent epidemiological data set the estimate as high as 500,000 patients (13, 14). Considering the growing number of DM patients in the country, the Croatian Ministry of Health has adopted the National Diabetes Program for the timeframe 2015 - 2020 and other strategic documents aiming to promote and facilitate early discovery and prevention of DM. The majority of preventive activities described in the aforementioned documents were intended to be implemented foremost in the primary healthcare sector, defined by the National Diabetes Program as the organizational and operational groundwork for all DM-related preventive actions (15). However, the diversion of resources during the COVID-19 epidemic may have affected the provision of routine healthcare services for DM patients, as well as their disease management capabilities.

The aim of this study was to investigate how the COVID-19 pandemic affected diabetes management and diabetes patients’ healthcare utilization patterns in Croatia.

## Materials and Methods

Various sources were used to extract the data on DM prevalence and incidence, the number of primary healthcare visits and hospitalizations labelled with ICD-codes pertaining to DM, and disease control indicators among DM patients in Croatia in the period 2017 - 2020. The Croatian national diabetes registry (CroDiab) and the Croatian hospitalization database (BSO) are administered by the Croatian Institute of Public Health, whereas the access to the Central Health Information System of the Republic of Croatia (CEZIH), containing information on all healthcare service provided by primary healthcare practitioners in Croatia, was granted to the Croatian Institute of Public Health by the Croatian Health Insurance Fund. The data on DM incidence and prevalence were extracted from the CroDiab registry. Data on DM-related primary healthcare visits were extracted by retrieving all records labelled with ICD-10 codes E10 - E14 in the period 2017 - 2020 from the CEZIH database. The data on DM-related hospitalizations were collected by extracting all records labelled with ICD-10 codes E10 - E14 in the same period from the BSO database. The data on DM control indicators in the studied period was extracted using disease control panels for DM patients, which are recorded in the CEZIH database.

A descriptive statistical data analysis was performed thereafter. The comparison of key disease control indicators (total cholesterol, fasting glucose, HbA1c %, and systolic blood pressure) across years 2017 - 2020 was performed using the Friedman nonparametric test. Crude prevalence, age-standardized prevalence and new cases’ rates were determined and the resulting data were standardized with regard to the European Standard Population (2013). The comparison of age-standardized rates across years and the determination of the ratio of age-standardized rates across years were performed using Smith’s formulas. The values of the empirical significance level p of 0.05 (p < 0.05) were considered statistically significant. The SPSS 21 software package was used for the data analysis.

## Results

The total number of patients registered in the CroDiab registry increased from 2017 until 2019 (297,298 registered patients in 2017; 307,200 in 2018; 313,625 in 2019). In 2020 the total number of registered patients decreased to 310,212. The number of new patients registered in the CroDiab registry displayed a slightly decreasing trend from 2017 until 2019 (39,414 in 2017; 37,987 in 2018; 36,749 in 2019); in 2020 it decreased sharply to 30,026.

The age-adjusted prevalence rate increased significantly from 2017 until 2019 (2017: 6,858/100,000; 2018: 7,053/100,000; 2019: 7,160/100,000). In 2020 age-adjusted prevalence rate was 7,088/100,000, but the decrease was insignificant compared to 2019. The age-adjusted rate of new cases decreased from 2017 until 2019 (2017: 910/100,000; 2018: 876/100,000; 2019: 845/100,000), with a significant decrease in 2020 (692/100,000) compared to 2019.

**Figure 1:**
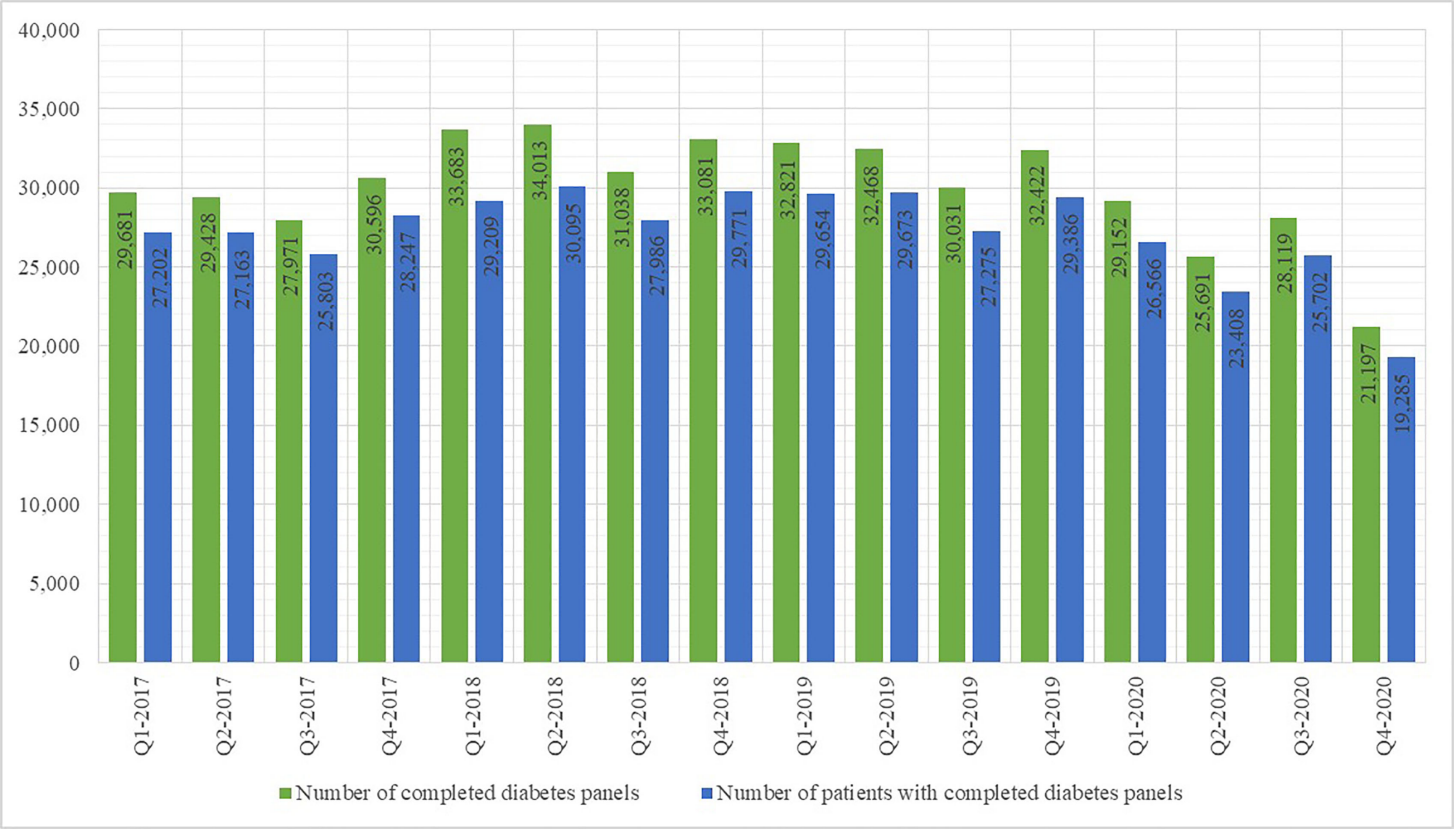
Number of completed diabetes control panels and patients with completed diabetes control panels per yearly quarter 2017 - 2020.

**Figure 2:**
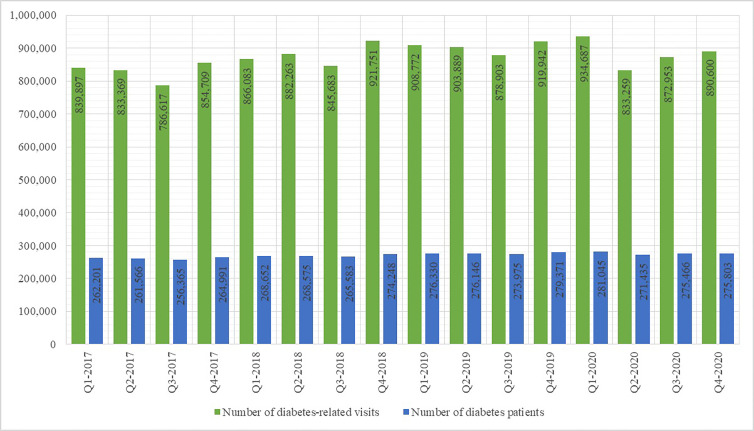
Number of diabetes-related primary healthcare visits per yearly quarter 2017 - 2020.

The number of DM control panels increased from 2017 (117,676) until 2018 (131,815), with a slight decrease in 2019 (127,742) and a sharp decrease in 2020 (104,159). A similar trend was observed regarding the numbers of patients with completed DM panels, DM-related visits to primary healthcare providers and DM patients who visited their primary healthcare provider. With particular regard to the number of completed DM panels and the number of DM-related visits to primary care providers per quarters, as shown in **Figures 1** and **2**, there was a significant decrease in Q2-2020 compared to Q1-2020.

**Figure 3:**
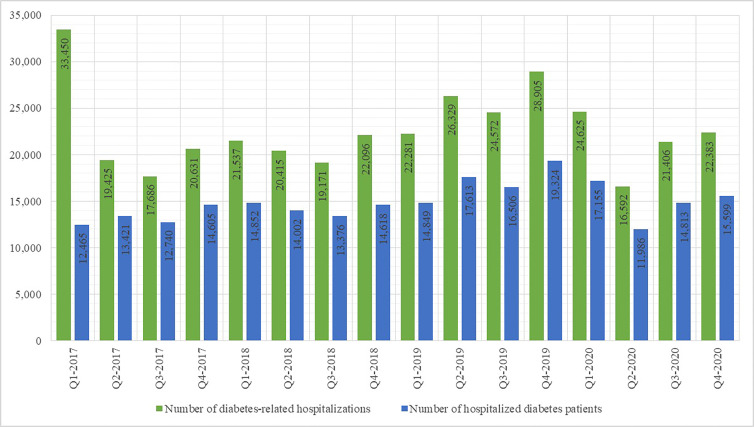
Number of diabetes-related hospitalisations and patients hospitalised for diabetes per yearly quarter 2017 - 2020.

A slightly different trend was observed regarding the number of DM-related hospitalizations, as shown in **Figure 3**. In 2017 there were 91,192 DM-related hospitalizations; the number of such hospitalizations decreased to 83,219 in 2018, increased again to 102,087 in 2019 and decreased to 85,006 in 2020. The number of patients undergoing DM-related hospital treatment displayed a similar tendency. As shown in **Figure 2**, the DM-related hospitalization rate during the second quarter of 2020 was nearly half the rate in the first quarter.

**Figure 4:**
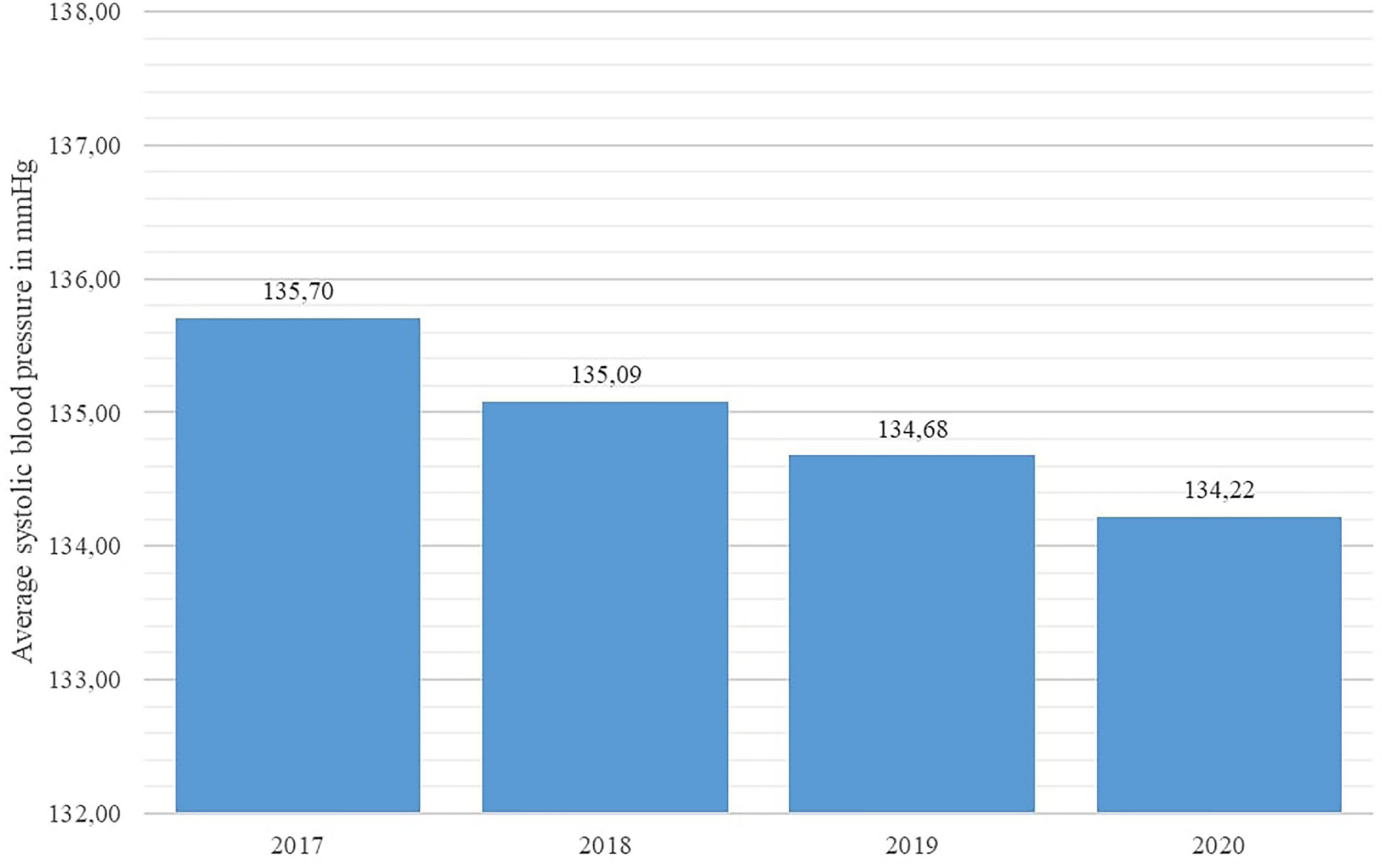
Average systolic blood pressure values in diabetes patients in Croatia per year, 2017 - 2020.

**Figure 5:**
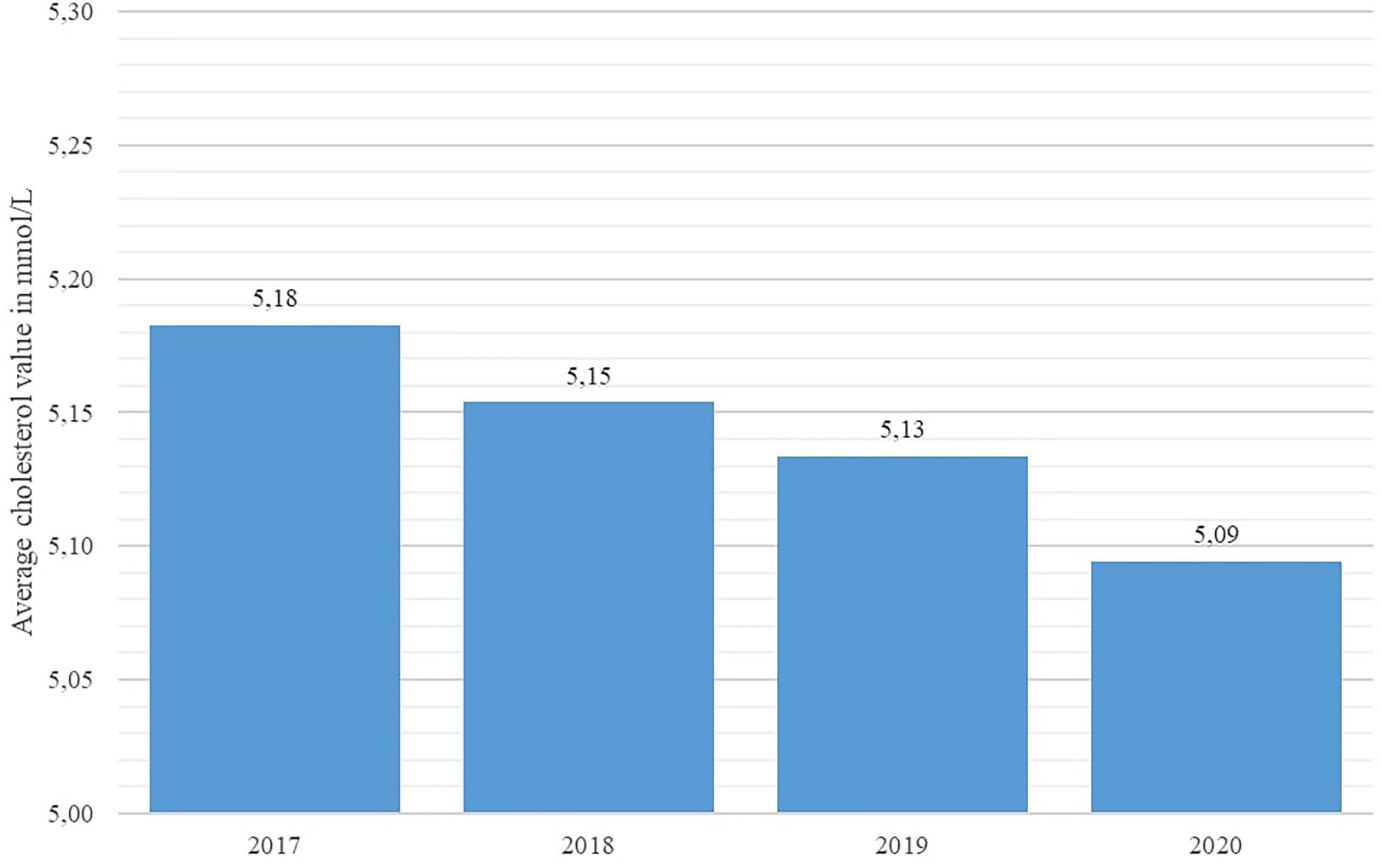
Average total cholesterol values in diabetes patients in Croatia per year, 2017 - 2020.

**Figure 6:**
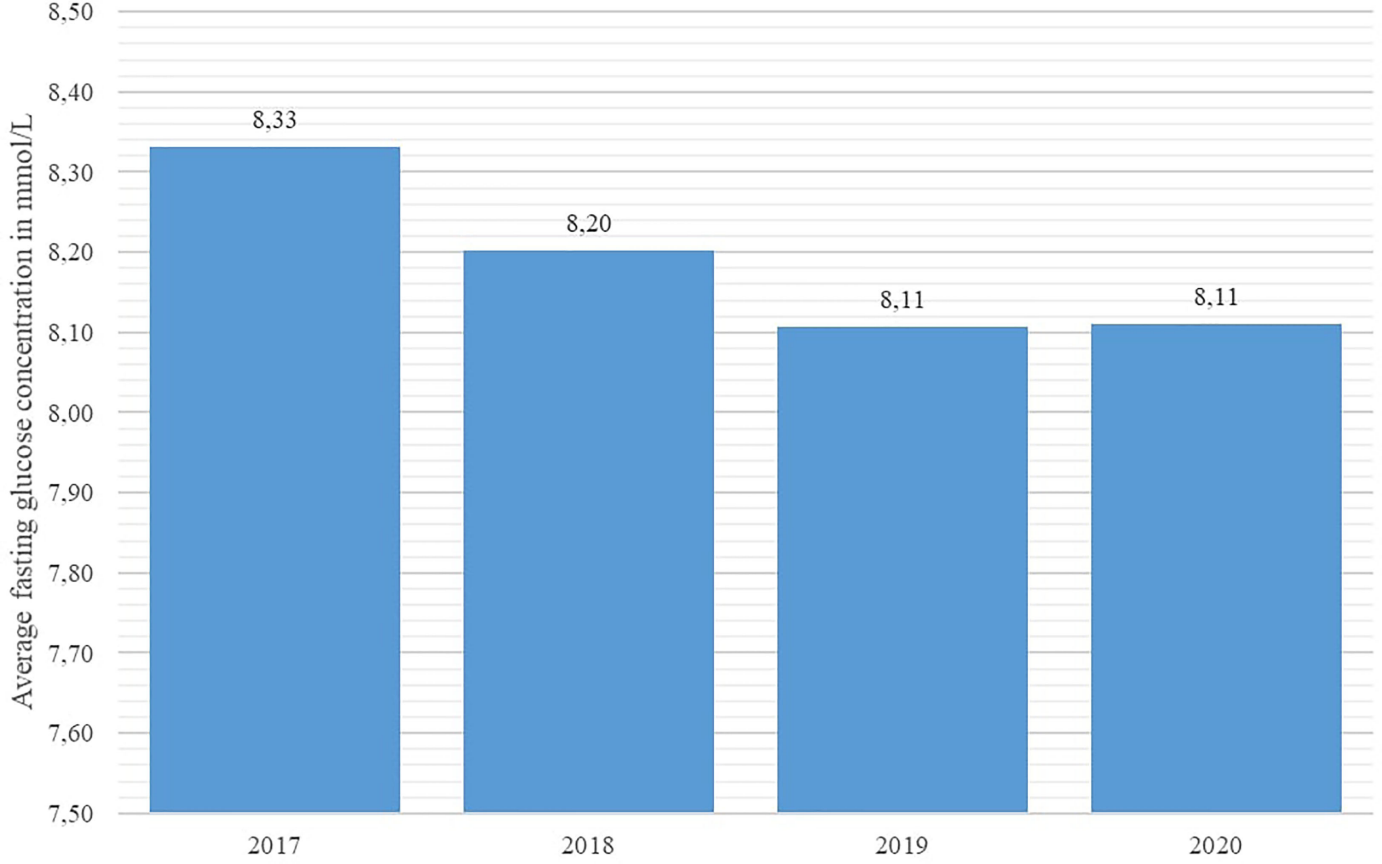
Average fasting glucose values in diabetes patients in Croatia per year, 2017 – 2020.

**Figure 7:**
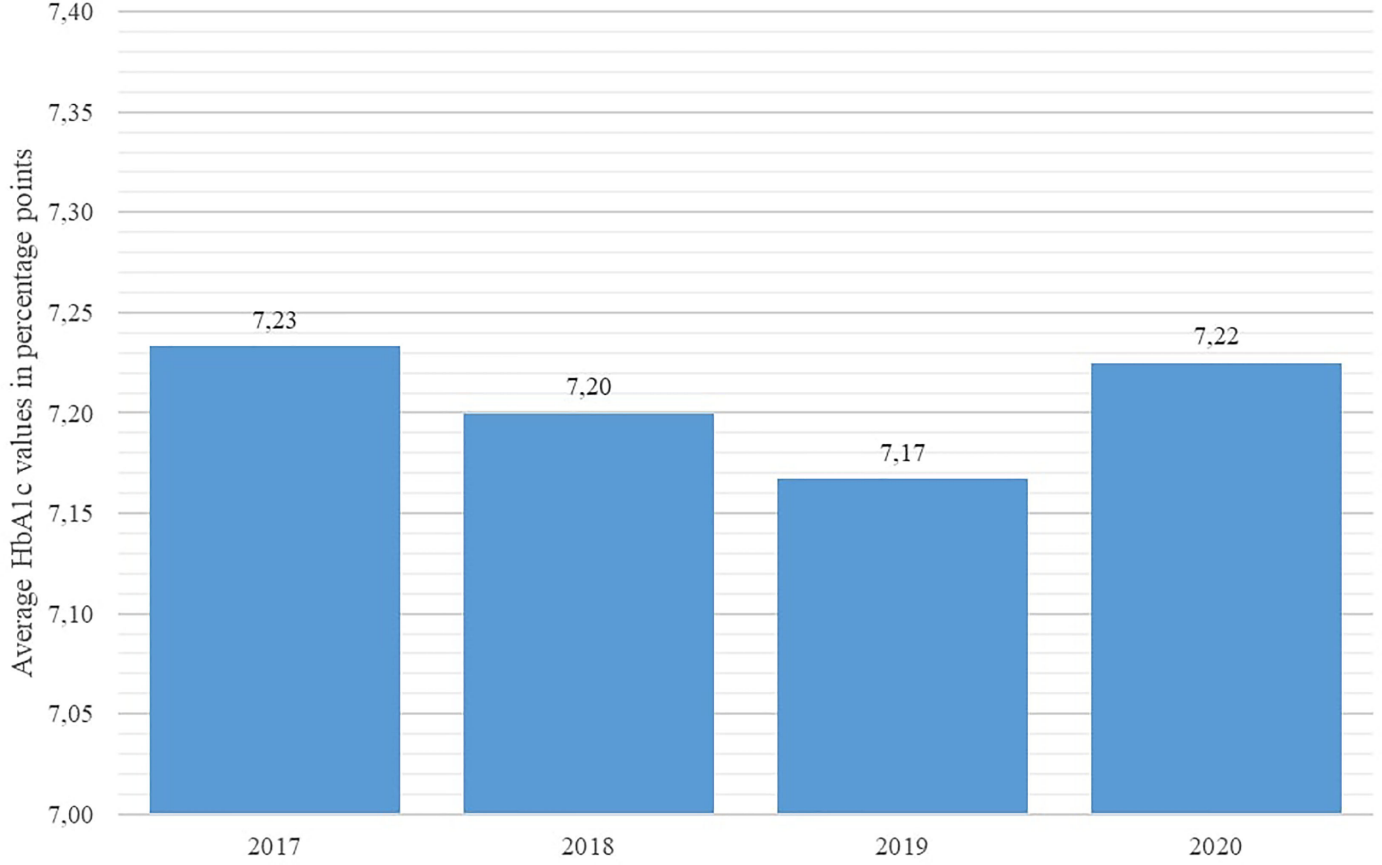
Average HbA1c percentages in diabetes patients in Croatia per year, 2017 - 2020.

Key disease control indicators (total cholesterol, fasting glucose, HbA1c %, and systolic blood pressure) were compared across the studied years using the Friedman nonparametric test. The test revealed statistically significant differences in all observed indicators (p < 0.001 for all indicators). As shown in **Figures 4** and **5**, average systolic blood pressure and total cholesterol values were continuously decreasing in observed period. Highest average fasting glucose values were recorded in 2017, whereas the lowest average values were recorded in 2019 and 2020 (**Figure 6**). However, average HbA1c % values were similar in 2017 and 2020, with the lowest average values recorded in 2019 (**Figure 7**).

## Discussion

The results of this study corroborate the hypothesized negative effect of the COVID-19 epidemic, the associated lockdowns and the disruptions to healthcare provision on both early disease discovery and disease management in DM patients. The number of newly-recorded DM patients in Croatia in 2020 was the lowest recorded in several years; in the absence of other factors which may influence the incidence of DM in the general population, the diminished accessibility of medical services to patients seeking healthcare for DM-related symptoms appears as a plausible explanation. The prevalence rate of DM in Croatia recorded its first decrease in years as well. Moreover, the DM-related hospitalization rate during the second quarter of 2020 was nearly half the rate in the first quarter, implying that a significant number of DM patients in need of stationary treatment (diagnostic or therapeutic) may have been left untreated. Regarding health monitoring provided to known DM patients, the number of disease control panels, comprising indicators such as glycaemia, HbA1c concentration etc., and provided by primary healthcare physicians, steadily decreased during the first two quarters of 2020, with a total reduction of approximately 20% in the second quarter of 2020 in comparison with the last quarter of 2019. Similar trends have been observed in other countries, such as United States and Japan (16, 17).

Besides aggravating the provision of healthcare, lockdowns associated with the COVID-19 pandemic may have been another detrimental factor for DM patients due to the suspension of recreational activities in communities and the general discouragement of outdoor activities possibly involving social contact by health authorities. Despite their efficiency in mitigating the transmission of COVID-19, such policies led large numbers of DM patients to spend a greater proportion of their time indoors, which in turn reflected on their physical activity patterns, as well as their diet and psychological condition. Instances of earlier research have associated disaster-related experiences in DM patients with a deterioration of disease control indicators such as HbA1c percentages (18–20). The results of research on DM disease control during the COVID-19 pandemic published so far further substantiate these findings (21, 22).

With regard to disease control indicators, this study revealed a steadily decreasing trend in both average cholesterol levels and systolic blood pressure values in studied individuals across the studied interval, including the year 2020. However, the average value of HbA1c % stagnated in 2020 in comparison with 2019 in the studied population, whereas the average fasting glucose concentration increased significantly in 2020 with regard to the previous year. This apparent inconsistency may be associated with the limited accessibility of healthcare services for DM patients, as a significant number of DM patients requiring healthcare services may have been prevented from attending their appointments due to lockdowns and travel restrictions, particularly patients with impaired physical mobility or those experiencing socio-economic deprivation; results of a recent Scottish study affirm this observation (23).

The chronic nature of diabetes mellitus (DM) as a metabolic disease and its pathophysiology require constant monitoring of glycaemia, as well as regular professional medical surveillance and care to prevent disease complications. The adverse effects of unregulated dysglycemia on the function of the human immune system have been well established, making DM one of the best-known risk factors for poor outcome in infectious diseases (24). A number of studies published heretofore has confirmed the association of poor COVID-19 clinical outcomes and previously existing chronic medical conditions, including DM (25–27). In this regard, a previous study on the association of comorbidities and COVID-19 disease outcomes in Croatia found DM patients to have significantly higher mechanical ventilation support and case fatality rates (28). Moreover, uncontrolled hyperglycemia has been found to have a negative effect on the immunological response consequent to COVID-19 vaccination, posing an additional risk for DM patients in case of COVID-19 infection (29).

Considering the somewhat unpredictable further evolution of the COVID-19 pandemic, new methods of providing healthcare to patients with chronic diseases will have to be investigated and put into use as soon as feasible to mitigate the effects of limited availability of medical services and patient mobility. Tele-health services designed in this respect for DM patients offer significant prospects for better disease control both in the short and long term (30). Furthermore, their implementation may decrease the costs of healthcare for DM patients, being of special importance due to the growing DM prevalence rate and enabling the diversion of financial resources to other segments of patient care.

The limitations of this study are related to the data sources used to conduct this study. All DM patients listed in the CroDiab registry have had their diagnosis confirmed by means of laboratory tests and relevant diagnostic criteria prerequisite for inclusion in the CroDiab patient registry. In some situations, however, ICD-10 codes relating to DM (E10 - E14) may have been erroneously attributed in patient records contained in CEZIH or BSO databases due to oversight or inadvertence. In this regard, diagnoses attributed during primary healthcare visits or hospital treatment might not necessarily correspond with the medical reason for the visit or hospitalization; however, the effect of misattribution errors is likely to be negligible. Furthermore, seasonal variation in the number of patient visits to primary healthcare providers and hospitalizations should be taken into account when analyzing healthcare utilization patterns, warranting future research to analyze longer timeframes to exclude any confounding factors.

The COVID-19 epidemic in Croatia compromised the provision of healthcare to all patients diagnosed with chronic illnesses, including DM patients. Reductions in the number of patient visits, patient consultations and completed DM control panels with primary healthcare providers were recorded, as well as reductions in numbers of DM-related hospitalizations. Considering the possible consequences of compromised disease control, further attention is necessary to accommodate the existing limitations of healthcare accessibility and provide for new methods to provide adequate DM patient care.

## Data Availability Statement

The data analyzed in this study is subject to the following licenses/restrictions: Datasets contain confidential patient information. Requests to access these datasets should be directed to IC, ivan.cerovecki@hzjz.hr.

## Author Contributions

IC and MŠ conceived and designed the study. MŠ acquired the data. IC and MŠ analyzed and interpreted the data. IC and MŠ drafted the manuscript. IC and MŠ critically revised the manuscript for important intellectual content. IC and MŠ gave approval of the version to be submitted; IC and MŠ agree to be accountable for all aspects of the work. All authors contributed to the article and approved the submitted version.

## Conflict of Interest

The authors declare that the research was conducted in the absence of any commercial or financial relationships that could be construed as a potential conflict of interest.

## Publisher’s Note

All claims expressed in this article are solely those of the authors and do not necessarily represent those of their affiliated organizations, or those of the publisher, the editors and the reviewers. Any product that may be evaluated in this article, or claim that may be made by its manufacturer, is not guaranteed or endorsed by the publisher.
